# A qualitative study to explore the symptoms and impacts of Crohn’s disease and to develop the Crohn’s Disease Diary

**DOI:** 10.1007/s11136-022-03233-9

**Published:** 2022-09-02

**Authors:** Rebecca Williams-Hall, Claire Trennery, Kate Sully, Samantha Wratten, Anya Francis, Dale Chandler, Jessica Flynn, Megan Turner, Daniel J. B. Marks, Alfred Sackeyfio, Marguerite Bracher, Alex Walker, Louise Walker-Nthenda, Rob Arbuckle, Tom Keeley

**Affiliations:** 1 Patient-Centered Outcomes, Adelphi Values, Bollington, Cheshire UK; 2 Value Evidence Outcomes, GlaxoSmithKline, Brentford, London, UK; 3 Value Evidence Outcomes, GlaxoSmithKline, Collegeville, PA USA; 4 Discovery Medicine, GlaxoSmithKline, Stevenage, Hertfordshire, UK; 5 Value Evidence Outcomes, GlaxoSmithKline, Stevenage, Hertfordshire, UK; 6 Clinical Sciences, GlaxoSmithKline, Brentford, London, UK

**Keywords:** Crohn’s disease, Patient-reported outcomes, Content validity, Symptoms, Instrument development

## Abstract

**Purpose:**

To explore symptoms and disease impacts of Crohn’s disease and to develop a new patient-reported outcomes (PRO) measure according to industry best practices.

**Methods:**

A conceptual model of relevant symptoms experienced by patients with Crohn’s disease was developed following a literature review. Three rounds of combined qualitative semi-structured concept elicitation and cognitive debriefing interviews with 36 patients (≥ 16 years) with Crohn’s disease and 4 clinicians were conducted to further explore the most commonly reported and most bothersome symptoms to patients. Interview results were used to update the conceptual model as well as items and response options included in The Crohn’s Disease Diary, a new PRO measure.

**Results:**

All patients (*N* = 36) reported abdominal pain, loose or liquid bowel movements, and high or increased frequency of bowel movements, with most reporting these symptoms spontaneously (100%, 92%, and 75%, respectively). All patients reported bowel movement urgency, but 61% reported this symptom only when probed. Most also reported that symptoms impacted activities of daily living, work/school, and emotional, social, and physical functioning (overall, 78%–100%; spontaneously, 79% – 92%). Data regarding core symptoms of Crohn’s disease from clinician concept elicitation interviews supported patient data. The 17-item Crohn’s Disease Diary assesses core symptoms and impacts of Crohn’s disease over 24 h, and extraintestinal manifestations over 7 days. The content validity of the diary was confirmed during cognitive debriefing interviews.

**Conclusion:**

The Crohn’s Disease Diary is a new PRO measure for the assessment of Crohn’s disease symptoms and impacts, developed according to industry best practices.

**Supplementary Information:**

The online version contains supplementary material available at 10.1007/s11136-022-03233-9.

## Plain English Summary

Questionnaires that measure symptoms and the impact of symptoms on patients’ quality of life are often used in clinical trials to measure how the severity of symptoms and the patient’s quality of life changes with treatment. These questionnaires should be developed based on feedback from patients and doctors to make sure they include the most important and relevant questions for patients.

The purpose of this study was to conduct patient and clinician interviews to better understand the main symptoms of Crohn’s disease and their impact on patients. Based on these interviews, a questionnaire called the Crohn’s Disease Diary was developed to assess the symptoms and impacts experienced by patients with Crohn’s disease.

The results of this study suggest that the Crohn’s Disease Diary includes relevant, easy-to-understand questions appropriate for use in future clinical trials to measure if patients’ symptoms and quality of life have improved with treatment.

## Introduction

Crohn’s disease is an inflammatory bowel disease (IBD) that is characterized by chronic inflammation of the gastrointestinal tract [1, 2]. Cardinal symptoms include abdominal pain and increased frequency, urgency, and loose consistency of stools [3–5]. Some patients also experience systemic symptoms such as fatigue, fever, or weight loss, and/or extraintestinal symptoms including inflammation of the eyes (uveitis, scleritis, and episcleritis), skin manifestations (pyoderma gangrenosum or erythema nodosum), or joint pain. Crohn’s disease has a progressive course for most patients, greatly impacting their quality of life [4]. Patients may experience psychological impacts such as depression or anxiety, fatigue, work disability and loss of productivity, or activity impairment [6]. Active disease is associated with lower quality of life, including impacts to social relationships and concerns about isolation [7, 8]. As such, both the U.S. Food and Drug Administration (FDA) and the European Medicines Agency (EMA) have expressed a desire for the reliable and valid measurement of symptoms and health-related quality of life (HRQoL) in Crohn’s disease clinical trials, and a number of patient-reported outcomes (PRO) measures have been used in this context [9, 10].

The Crohn’s Disease Activity Index (CDAI), which uses a weighted scoring algorithm to combine patient-reported symptoms and a general well-being rating with clinical assessments, was originally developed in 1976 and has traditionally been used in clinical trials [11–13]. Another measure, the inflammatory bowel disease questionnaire (IBDQ), was developed in 1989 and has also been widely used in clinical trials [14]. However, both were developed prior to the release of FDA best practice PRO guidance and neither meets current guidelines [15]. Therefore, the FDA and EMA have encouraged sponsors to consider these recommendations when developing measurement strategies for clinical trials. Thus, the objectives of this study were to better understand the patient experience of Crohn’s disease from the patient and clinician perspective, providing essential information to researchers developing measurement strategies in this therapy area. The study also aimed to understand the core symptoms of Crohn’s disease, and the impact of these on HRQoL. Additionally, a daily diary to robustly measure the core symptoms and impacts experienced by patients with Crohn’s disease was developed.

## Methods

### Study design

**Fig. 1:**
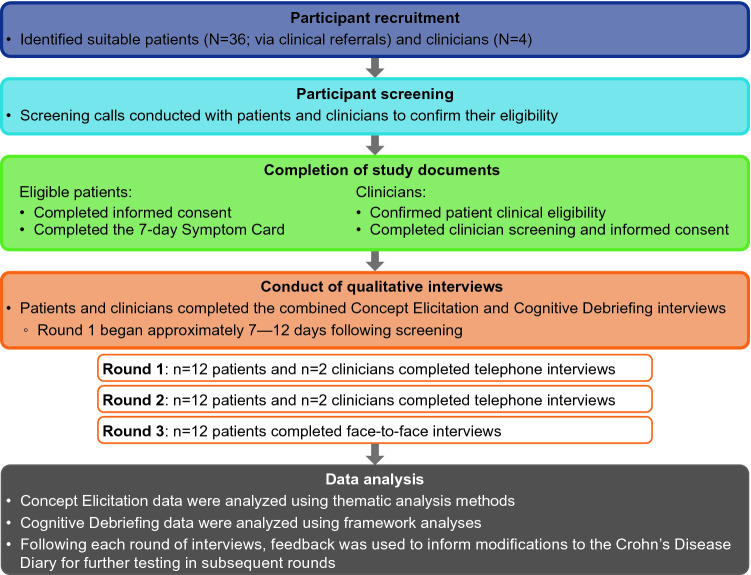
Study design

This was a cross-sectional, qualitative interview study in the USA, involving 3 rounds of semi-structured combined concept elicitation and cognitive debriefing interviews (Fig. 1) conducted between March and September 2019. Before the first round of interviews, a targeted review of literature was conducted. Articles, conference abstracts, and studies on the patient experience of Crohn’s disease published between 2008 and 2018 were used to develop an initial conceptual model to describe signs and symptoms of Crohn’s disease. Based on the literature review, a draft conceptual model and preliminary 19-item daily symptoms diary was developed for the study. Items included in the 19-item diary represented key symptoms and impacts of Crohn’s disease (including extraintestinal symptoms). Response options were item specific, depending on the type of rating scale considered most appropriate for each item (e.g., 0–10 numerical scales or Likert scales). Patients were asked to recall key symptoms and impacts over the past 24 h and extraintestinal symptoms over the past 7 days.

The study was approved by an independent scientific review committee (The Copernicus Group, Cary, NC; IRB tracking number: 20190635, IRB amendment tracking number 20190635). All patients and clinicians provided oral and written informed consent prior to participating in any study-related activities. Written permission was obtained from the parents/guardians of adolescent patients and written assent was obtained from the adolescents.

### Study population

Medquest Global (Valencia, CA), a third-party recruitment agency assisted with the recruitment of patients from four geographically diverse locations in the USA (New Orleans, Louisiana; Baltimore, Maryland; Los Angeles, California; and Chicago, Illinois). Patients were recruited via referrals from gastroenterologists and general practitioners. Age, sex, race, education, disease severity, and treatment sampling quotas were applied to the sample patient population to ensure a range of demographic and clinical characteristics. Sample size was determined based on the principles of conceptual saturation. It was estimated that a total of 36 patients (*n* = 12 for each round of interviews) would be adequate to achieve saturation and to ensure sufficient clinical and demographic diversity in each round of interviews [16–18].

Adolescent (≥ 16 years of age) and adult patients with radiographically, histologically, and/or endoscopically confirmed Crohn’s disease, diagnosed ≤ 6 months prior to screening, who had experienced symptoms within the 3 months prior to the study were eligible. Severity of disease was based on patient-reported data collected via a 7-day diary card completed prior to study entry. Disease severity categories and detailed eligibility criteria are included in the Supplemental Materials.

Clinicians specializing in the management or treatment of adult and/or adolescent patients with Crohn’s disease on a regular basis (i.e., at least 8 patients/month) were eligible. Experience treating patients with biologic agents, fluency in spoken and written English, and access to a telephone or computer was also required.

### Qualitative interviews

The first and second rounds of combined concept elicitation and cognitive debriefing interviews were conducted by trained Adelphi Values interviewers via telephone and the third was face to face. Interviews were scheduled 7 to 12 days after screening. Each interview lasted approximately 90 min, with breaks offered to participants as needed. The time devoted to CE versus CD varied across the rounds, with greater focus on CE in round 1 (~ 40 min). To allow more focus on CD in rounds 2 and 3, less time was spent on CE (~ 20–25 min). In the concept elicitation portion, broad open-ended interview questions were posed in an unbiased manner to explore spontaneously reported patient experiences of Crohn’s disease. For example, interviewers asked patients to describe their experience of Crohn’s disease. These spontaneously reported symptoms likely reflect those which patients attribute most to their experience of Crohn’s disease, but to ensure that the full range of potentially applicable symptoms (as identified through literature review and clinician input) are explored, additional probing questions by interviewers are necessary. Thus, following the open-ended questions, more focused questions probed for topics of interest not spontaneously mentioned. Patient-friendly language was used when probing for symptoms. For example, patients were asked about the concept of urgency, which was commonly described as “needing to use the bathroom immediately” or “rushing to a toilet.” Similarly, to explore the concept of tenesmus, patients were asked about a “sensation of needing to go to the toilet but not passing anything.”

In the cognitive debriefing portion of the interview, patients participated in a “think-aloud exercise” during which they read and responded to the items in the daily symptoms diary, providing thoughts and opinions on each item. Patients were also asked detailed questions about item relevance, item wording, instructions, recall period, response options, ease of completing the items, and usability of the diary in an electronic PRO (ePRO) format.

Following each round of interviews, feedback from the concept elicitation portion determined if content modifications to the conceptual model and the daily symptoms diary were needed. Based on feedback from the cognitive debriefing portion of the interviews, patient understanding of the instructions, items, response options, and recall periods in the daily symptoms diary was assessed and wording could be modified for clarity. Additionally, item relevance and overlap among items was assessed to determine if items should be added or deleted prior to the next round of interviews. Items retained in the final daily symptoms diary (Crohn’s Disease Diary) also underwent a translatability assessment.

In addition to patient interviews, combined concept elicitation and cognitive debriefing qualitative telephone interviews were conducted with clinicians to gain clinical insights into the patient experience of Crohn’s disease in terms of symptom presentation and impact on daily life, as well as to provide feedback for the items included in the daily symptoms diary. Broad, open-ended questions were first used to prompt spontaneous responses, followed by more focused questions to probe on topics of interest or for clarification. The interviews were conducted in two rounds corresponding to the first two rounds of patient interviews.

### Data analysis

All interviews were audio-recorded and transcribed verbatim. Data from adolescents (*n* = 4 for all rounds of interviews) and adults (*n* = 32) were pooled, as no conceptual differences between the age groups were expected or identified during analysis. Qualitative analysis of patient and clinician interview transcripts was conducted using ATLAS.ti version 7.2013 (Scientific Software Development GmbH B, Germany).

Coding schemes were derived for the interview transcripts using the first two transcripts. These schemes were used throughout the analysis to ensure consistent application and grouping of codes across transcripts. The concept elicitation sections of transcripts were analyzed using thematic analysis. Participant quotes that pertained to symptoms, patient experience, or patient impact of Crohn’s disease, and whether these were reported spontaneously or when probed, were assigned corresponding concept codes. The cognitive debriefing sections of transcripts were analyzed using a framework approach. Dichotomous codes were assigned to each item, instruction, response option(s), and recall period to indicate relevance, understanding, or difficulty to complete. Codes were also used to indicate why each response option was chosen and how the content of each item applied to the patient experience, as well as to provide suggestions for item, instruction, or response option wording or formatting changes, and general feedback on the daily symptoms diary. An induction-abduction approach was taken to identify themes in the data [19].

To ascertain whether conceptual saturation was achieved, transcripts were ordered chronologically, divided into four groups of 9 patients each and findings from each group of interviews were compared in an iterative fashion. A conceptual saturation grid was developed to summarize results and to determine if conceptual saturation had been achieved. Saturation was deemed to be achieved when no new concepts emerged in the last (fourth) group of interviews.

## Results

### Demographic and clinical characteristics

A total of 36 patients (*n* = 12 in each round) were interviewed and all sampling quotas were met. The mean age was 40.5 years (range: 16–74 years), there were slightly more females (21/36 [58%]) than males, and most patients were White/Caucasian/European (24/36 [67%]) (Table 1). Nearly two-thirds (22/36 [61%]) of patients were categorized as having moderate to severe Crohn’s disease. Time since diagnosis ranged from 6 months to 20 years and the sample was diverse in terms of location of disease (ileum, 25/36 [69%]; proximal small bowel 22/36 [61%]; colon, 22/36 [61%]; esophagus/stomach, 27/36 [44%]).

**Table 1:** Patient demographics and clinical characteristics

Patient characteristic	Round 1 (*n* = 12)	Round 2 (*n* = 12)	Round 3 (*n* = 12)	Total (*N* = 36)
Age (years), *n* (%)				
0–18	1 (8)	2 (17)	1 (8)	4 (11)
19–25	2 (17)	2 (17)	1 (8)	5 (14)
26–35	3 (25)	3 (25)	2 (17)	8 (22)
36–45	3 (25)	1 (8)	0 (0)	4 (11)
46–50	2 (17)	0 (0)	2 (17)	4 (11)
51–59	0 (0)	2 (17)	3 (25)	5 (14)
60 +	1 (8)	2 (17)	3 (25)	6 (17)
Sex, *n* (%)				
Female	6 (50)	8 (67)	7 (58)	21 (58)
Male	6 (50)	4 (33)	5 (42)	15 (42)
Ethnicity, *n* (%)				
Not Hispanic or Latino	12 (100)	10 (83)	8 (67)	30 (83)
Hispanic or Latino	0 (0)	2 (17)	4 (33)	6 (17)
Race, *n* (%)				
White/Caucasian/European	9 (75)	5 (42)	10 (83)	24 (67)
African American/African heritage	3 (25)	7 (58)	0 (0)	10 (28)
White Arabic/North African heritage	0	0	1 (8)	1 (3)
Other–Hispanic	0	0	1 (8)	1 (3)
Educational level, *n* (%)				
Some years of college	3 (25)	4 (33)	3 (25)	10 (28)
University or college degree (2 or 4 years)	4 (33)	1 (8)	4 (33)	9 (25)
High school diploma or GED	0 (0)	5 (42)	3 (25)	8 (22)
Some high school	1 (8)	2 (17)	1 (8)	4 (11)
In college now	2 (17)	0 (0)	0 (0)	2 (6)
Certificate program	1 (8)	0 (0)	1 (8)	2 (6)
Graduate or professional degree	1 (8)	0 (0)	0 (0)	1 (3)
Work status, *n* (%)				
Working full- or part-time	8 (67)	7 (58)	10 (83)	25 (69)
Full-time homeworker	2 (17)	1 (8)	0 (0)	3 (8)
Not applicable	0 (0)	2 (17)	0 (0)	2 (6)
Not working due to Crohn’s disease	1 (8)	0 (0)	0 (0)	1 (3)
Other–student	1 (8)	0 (0)	1 (8)	2 (6)
Looking for work	0 (0)	1 (8)	1 (8)	2 (6)
Retired	0 (0)	1 (8)	0 (0)	1 (3)
Severity level, *n* (%)				
Moderate to severe	5 (42)	11 (92)	6 (50)	22 (61)
Remission to mild	7 (58)	1 (8)	6 (50)	14 (39)
Time since diagnosis in months, *n* (%)				
121–240	5 (42)	2 (17)	2 (17)	9 (25)
61–120	2 (17)	4 (33)	3 (25)	9 (25)
25–60	3 (25)	4 (33)	6 (50)	13 (36)
13–24	1 (8)	2 (17)	1 (8)	4 (11)
6–12	1 (8)	0 (0)	0 (0)	1 (3)
How diagnosis was confirmed,^a^ *n* (%)				
Endoscopy	12 (100)	8 (67)	9 (75)	29 (81)
Histology	10 (83)	5 (42)	6 (50)	21 (58)
Radiology	7 (58)	8 (67)	3 (25)	18 (36)
Location of patients’ disease,^a^ *n* (%)				
Ileum^b^	10 (83)	6 (50)	9 (75)	25 (69)
Proximal small bowel	3 (25)	11 (92)	8 (67)	22 (61)
Colon**	8 (67)	5 (42)	9 (75)	22 (61)
Esophagus and/or stomach	2 (17)	9 (75)	5 (42)	16 (44)
Perianal disease	1 (8)	2 (17)	0 (0)	3 (8)
Oral cavity	0 (0)	0 (0)	1 (8)	1 (3)
Treatment experience, *n* (%)				
Biologic-naïve	4 (33)	7 (58)	5 (42)	16 (44)
Biologic-experienced	8 (67)	5 (42)	7 (58)	20 (56)
Comorbid conditions, *n* (%)				
No	10 (83)	8 (67)	9 (75)	27 (75)
Yes^c^	2 (17)	4 (33)	3 (25)	9 (25)

^a^27 patients had  >1 method of diagnosis; 28 patients had >1 disease location

^b^18 patients experienced both ileal and colonic disease (*n* = 18); 3 patients had only ileal or only colonic disease

^c^Eye disorder (*n* = 1), depression (*n* = 3), anemia (*n* = 1), diabetes mellitus type 2 (*n* = 2), endometriosis (*n* = 1), hypertension (*n* = 1), gastroesophageal reflux disease (*n* = 1), asthma (*n* = 1)

A total of 4 clinicians (round 1, *n* = 2; round 2, *n* = 2) were interviewed. Three clinicians had > 20 years of experience treating Crohn’s disease, one had 6 years, and all reported seeing 10 to 40 patients with Crohn’s disease each month.

### Patient concept elicitation

**Fig. 2:**
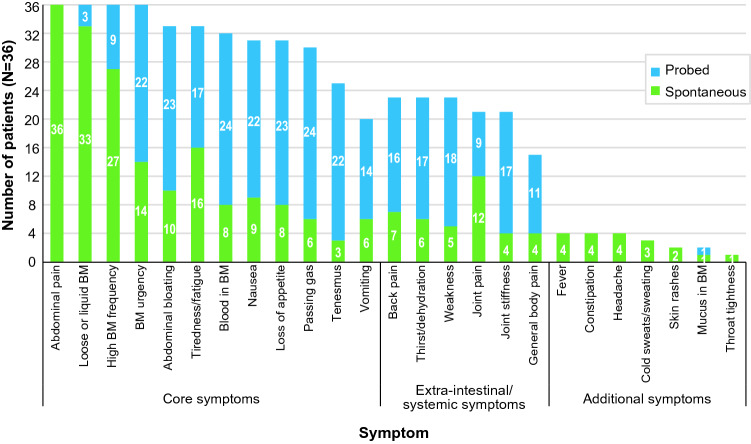
Number of patients reporting each symptom spontaneously or probed (N = 36). *BM*, bowel movement

The frequency with which patients reported symptoms spontaneously versus probed is shown in Fig. 2. Patients (*N* = 36) reported a total of 25 symptoms, broadly categorized as 12 core symptoms (bowel and abdominal symptoms), 6 extraintestinal symptoms, and 7 additional symptoms (Fig. 2**)**. Every patient reported that they experienced abdominal pain, loose or liquid bowel movements, high or increased frequency of bowel movements, and bowel movement urgency related to Crohn’s disease, either spontaneously or after being probed. Abdominal pain was reported spontaneously by all patients. Most reported loose or liquid bowel movements (33/36 [91.7%]) and high or increased frequency of bowel movements (27/36 [75.0%]) spontaneously, whereas the majority reported bowel movement urgency only after being probed (22/36 [61%]). Examples of how the most frequently reported symptoms were described by individual patients include:

Patient 1: Currently I am having a lot of loose stools.
Patient 2: I have a lot of stomach cramps, and sometimes they actually take me to the ground.
Patient 1: I mean there are days that I can go to the bathroom like 10-15 times a day…probably at least once a week.

**Fig. 3:**
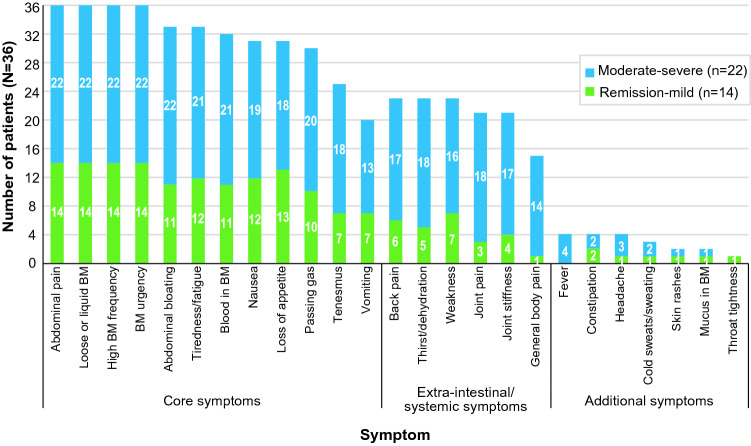
Number of patients reporting each symptom by Crohn’s disease severity (*N* = 36). *BM*, bowel movement

Although most symptoms were reported with similar frequency across disease severity groups, tenesmus, back pain, thirst/dehydration, weakness, joint pain, joint stiffness, and general body pain were reported more by patients with moderate to severe disease (Fig. 3). Fever and throat tightness were reported only by patients with moderate to severe disease. Examples of quotes from individual patients with moderate to severe disease include:

Patient 3: With the joint pain it lasts for like a couple days.
Patient 4: I feel like, like I want to drink a lot of water.

**Fig. 4:**
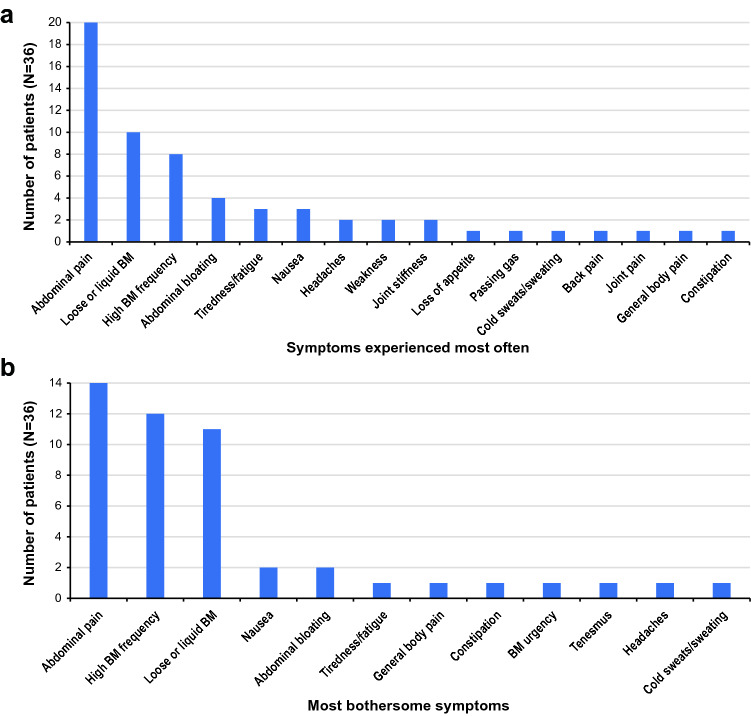
**A** Symptoms experienced most often by patients and **B** Most bothersome symptoms (*N* = 36)^a^. ^a^Total number of symptoms exceeds the number of patients since some patients reported >1 symptom. *BM*, bowel movement

The 3 symptoms that patients experienced most often (abdominal pain, loose or liquid bowel movements, and high frequency of bowel movements) were also reported to be the most bothersome and most important to change in response to treatment (Fig. 4).

Patients also reported the impact of their disease on HRQoL. All patients, either spontaneously (33/36 [92%]) or probed (3/36 [8%]), reported that activities of daily living (ADL) were impacted by Crohn’s disease symptoms. Most patients (34/36 [94%]) reported emotional impacts, most commonly feeling worried or anxious about urgently needing to have a bowel movement. Work/school impacts were reported by 33/36 (92%), including the need for frequent bathroom breaks. Most patients (32/36 [89%]) reported that their social functioning was impacted, particularly when taking vacations or going to restaurants, and that the quality of social relationships was impacted (11/32 [34%]), including feelings of isolation and effects on sexual relationships. Physical functioning impacts were reported by 28/36 (78%), with difficulty carrying out sports/exercise reported most frequently. Most patients reported emotional, work/school, social, and physical impacts spontaneously (79% − 88%). Sleep was also affected for most (92%), but only 42% reported this spontaneously. Examples of quotes from individual patients describing the impact of their disease on HRQoL are as follows:

Patient 5: Just like doing household chores and stuff…I don’t really feel like I have the energy to do it.
Patient 6: I'm always looking for the bathroom, always wondering when it's going to hit…I love to go fishing and there's no restroom outside.
Patient 7: I feel hopeless. Like I feel like there's nothing that I can do. I feel like life is being taken away from me because I can't do anything.

Two-thirds of patients spontaneously reported additional impacts that did not fit neatly into the general HRQoL categories above. Weight loss was reported by 12/24 (50%) (most frequently attributed to loss of appetite [5/12, 42%]), 8/24 (33%) reported fecal incontinence as an impact of bowel urgency, and 7/24 (29%) reported the need to lie down or rest when symptoms were severe. A small number of patients spontaneously reported rectal discomfort due to loose stools (5/24 [21%]), while others reported financial impacts (4/24 [17%]), including the cost of purchasing products required due to symptoms and lost income due to time off work.

The conceptual saturation grid was reviewed following the last (fourth) group of interviews. All symptoms emerged spontaneously within the first two groups of interviews and all impact domains emerged spontaneously in the first group of interviews, indicating that saturation was achieved.

### Patient perception of flares and remission

Based on the initial conceptual model from the literature review, the concept of a “flare” is well recognized. Although not consistently defined, in clinical practice it is generally understood to mean a period during which disease symptoms are more active. Twenty-six patients were asked to describe their general experience of a flare. Some patients (5/26 [19%]) defined a flare as a general worsening of symptoms, some (12/26 [46%]) reported that abdominal pain increased, and others said that more frequent/severe loose or liquid bowel movements (5/26 [19%]) and more frequent bowel movements (4/26 [15%]) were indicative of a flare. Small numbers of patients (1 − 2 for each symptom) reported that the emergence of new symptoms such as bloating, fever, cold sweats/sweating, tiredness/fatigue, back pain, nausea, loss of appetite, skin rashes, or dehydration denoted a flare.

In the third round of interviews, patients (*n* = 12) were asked what the term “remission” meant to them. Eight patients had heard of the term and responses were variable, ranging from having a reduction in symptoms to having no symptoms or feeling normal. Responses also varied when patients were asked which symptoms would need to improve to consider themselves in remission, including a reduction in loose or liquid stools, urgency, or abdominal or joint pain.

### Physician concept elicitation

Clinicians (*N* = 4) reported a total of 23 symptoms typically experienced by patients with Crohn’s disease. Symptoms most frequently reported by patients (abdominal pain, liquid bowel movements, high or increased frequency of bowel movements, and bowel movement urgency) were also reported by all clinicians. Patient-reported core symptoms and extraintestinal symptoms were also reported by physicians, whereas oral ulcers, vision changes, and vitamin absorption issues were each reported only by 1 clinician. Clinicians did not report fever, constipation, headache, cold sweats/sweating, mucus in stools, or throat tightness, whereas these symptoms were reported by patients. Clinicians also reported a relationship between disease location and symptoms. Two clinicians reported that patients with small bowel disease frequently present with bloating, especially after a high-fiber meal. Another clinician reported that patients with distal colonic inflammation or rectal involvement frequently present with tenesmus.

Clinician-reported data were generally similar to patient data regarding which symptoms were the most bothersome and most important to treat. In addition, clinicians emphasized the importance of recognizing fatigue as a symptom of Crohn’s disease. Consistent with results from patient interviews, clinicians reported that Crohn’s disease greatly impacts HRQoL.

### Conceptual model

**Fig. 5:**
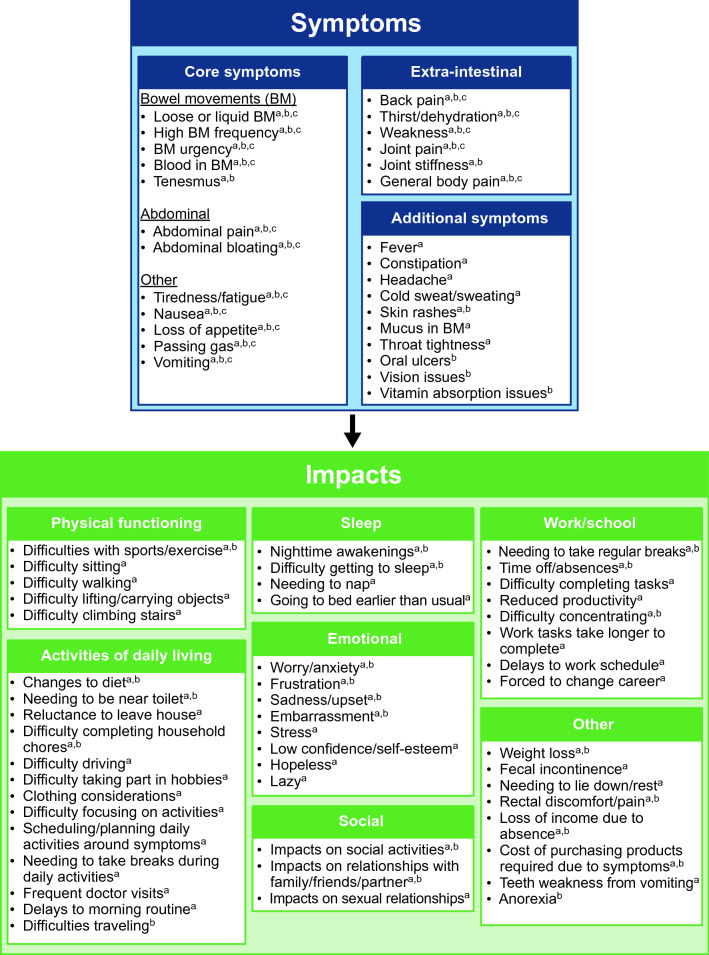
Conceptual model. ^a^Present in patient interviews (*N* = 36). ^b^Present in clinician interviews (*N* = 4). ^c^Present in preliminary conceptual model based on literature review

Based on patient and clinician concept elicitation results, the conceptual model, previously informed by the literature review alone, was updated (Fig. 5). The updated model includes additional relevant and important symptoms reported during the concept elicitation portion of the interviews that were not included in the original model. These included patient-reported symptoms such as fever, constipation, headache, cold sweats/sweating, mucus in stool, and throat tightness; and clinician-reported symptoms such as oral ulcers, vision issues, and vitamin absorption issues. Tenesmus, joint stiffness, and skin rashes, reported by both patients and clinicians, were also added. There was more of a disparity between patient- and clinician-reported HRQoL impacts. Patient-reported impacts added to the model included ADLs such as reluctance to leave the house and difficulty driving, as well as emotional, physical, and work/school impacts not reported by physicians (e.g., stress, difficulty sitting, and difficulty completing work tasks).

### Crohn’s Disease Diary

The preliminary 19-item daily symptoms diary was based on the literature review and the initial conceptual model. Development of the final version of the Crohn’s Disease Diary was in accordance with industry guidelines and includes items generated from the target population [9, 13]. Following three rounds of combined concept elicitation and cognitive debriefing interviews, a total of 17 items (using patient-friendly language) and 2 instructions (highlighting the recall period of the subsequent items) were retained in the final version of the Crohn’s Disease Diary (Table 2). Response options are item specific, including numerical frequency rating scales, severity scales, yes/no options for presence/absence of a symptom, or “select all that apply.” The first 11 items assess core symptoms and impacts of Crohn’s disease with a 24-h recall period, including bowel movement frequency, loose or liquid stool, blood in stool, volume of blood, urgency, tenesmus, abdominal pain, bloating, nausea, fatigue, and sleep disturbance. The remaining items have a 7-day recall period and pertain to extraintestinal manifestations, including items to assess the presence and severity of joint pain and stiffness in different locations.

**Table 2:** Items debriefed between interviews and retained in the Crohn’s Disease Diary

Symptom/impact	Preliminary item	Changes to item	Final item (changes to item shown in bold text)
Frequency of bowel movements	In the past 24 h, how many times did you go to the toilet where you had a bowel movement?	The definition of bowel movement was refined based on patient and clinician feedback in round 2	In the past 24 h, how many times did you go to the toilet where you had a bowel movement?
	A bowel movement is defined as when you pass a stool (solid, loose, and/or liquid) and/or blood or mucus		A bowel movement is defined as when you pass a stool (solid, loose, and/or liquid). *A bowel movement could include blood and/or mucus. It could also be blood and/or mucus alone*
Loose or liquid bowel movements	In the past 24 h, how many times did you go to the toilet where you had a bowel movement which was loose or liquid?		In the past 24 h, how many times did you go to the toilet where you had a bowel movement which was loose or liquid?^a^
			*A loose or liquid bowel movement could include blood and/or mucus. It could also be blood and/or mucus alone*
Blood in bowel movements	In the past 24 h, how many times did you go to the toilet where you had a bowel movement that had blood in it?	Based on patient feedback in round 3, clarification was made to the item stem to include blood alone as a bowel movement	In the past 24 h, how many times did you go to the toilet where you had a bowel movement that had blood in it? *This includes if you passed blood alone.*^a^
Volume of blood	Please rate the amount of blood in your bowel movement(s) at its worst in the past 24 h	Response options to further define the amount of blood were added based on patient feedback in round 1	Please rate the amount of blood in your bowel movement(s) at its worst in the past 24 h
		Although some patients suggested adding a “no blood” item, it was determined that this would not be necessary since patients would be able to skip the item within the ePRO platform if they had reported having no blood in their bowel movements	*[A little blood / Some blood / A lot of blood]*
Urgency	In the past 24 h, how many times did you have to rush to the toilet for a bowel movement?	No changes recommended	In the past 24 h, how many times did you have to rush to the toilet for a bowel movement?
Tenesmus	In the past 24 h, did you have a feeling that you needed to pass a bowel movement, but you did not pass anything?	No changes recommended	In the past 24 h, did you have a feeling that you needed to pass a bowel movement, but you did not pass anything?^a^
Abdominal pain	Please rate your worst abdominal pain in the past 24 h	Based on feedback from patients in round 1, abdominal pain was further defined to include cramps	Please rate your worst abdominal pain in the past 24 h
	[None, Mild, Moderate, Severe]	Response options were changed to a 0 to 10 scale due to patient preference in round 3	*Abdominal pain could include cramps*
			*[0 to 10 scale]*
Abdominal bloating	Please rate your worst abdominal pain in the past 24 h	No changes recommended	Please rate your worst abdominal pain in the past 24 h
	[0 to 10 scale]		[0 to 10 scale]
Nausea	Please rate your worst nausea (feeling like you need to throw up) in the past 24 h^b^	No changes recommended	Please rate your worst nausea (feeling like you need to throw up) in the past 24 h
	[0 to 10 scale]		[0 to 10 scale]
Fatigue/tiredness	Please rate your worst fatigue/tiredness in the past 24 h	No changes recommended	Please rate your worst fatigue/tiredness in the past 24 h
	[0 to 10 scale]		[0 to 10 scale]
Sleep disturbances	How many times did you wake up during the night to have a bowel movement?	Due to consistency with other items, the recall period was added to the item stem	How many times did you wake up *in the past 24 h* to have a bowel movement?
Extraintestinal symptoms	In the past 7 days, did you experience any of the following? (Check all that apply)	Due to the perceived need to assess back stiffness, this was added to the response options	In the past 7 days, did you experience any of the following? (Check all that apply)
	Back pain, General body pain, Joint pain, Joint stiffness	Although some patients suggested adding an “other” category, it was determined that this would not be necessary since response options can be left blank within the ePRO platform	Back pain, General body pain, Joint pain, *Back***/**Joint stiffness
Back pain	Please rate your worst back pain in the past 7 days	No changes recommended	Please rate your worst back pain in the past 7 days
	[0 (no back pain)—10 (extreme back pain)]		[0 (no back pain)—10 (extreme back pain)]
Joint pain location	Please indicate the joints in which you have experienced pain in the past 7 days? Please select all that apply	Based on the translatability assessment, the item stem was clarified	Please indicate *in which of your joints you experienced* pain in the past 7 days. Please select all that apply
	[Hips, Knees, Ankles, Feet/toes, Shoulders, Neck, Elbows, Wrists, Hands/fingers]		[Hips, Knees, Ankles, Feet/toes, Shoulders, Neck, Elbows, Wrists, Hands/fingers]
Worst joint pain	Please rate your worst joint pain in the past 7 days	No changes recommended	Please rate your worst joint pain in the past 7 days
	[0 (no joint pain)—10 (extreme joint pain)]		[0 (no joint pain)—10 (extreme joint pain)]
Morning joint stiffness location	Please indicate the joints in which you have experienced morning stiffness in the past 7 days? Please select all that apply	Based on the translatability assessment, the item stem was clarified	Please indicate *in which of your joints you experienced* morning stiffness in the past 7 days. Please select all that apply
	[Hips, Knees, Ankles, Feet/toes, Back, Shoulders, Neck, Elbows, Wrists, Hands/fingers]		[Hips, Knees, Ankles, Feet/toes, Back, Shoulders, Neck, Elbows, Wrists, Hands/fingers]
Worst morning joint stiffness	Please rate your worst morning joint stiffness in the past 7 days	No changes recommended	Please rate your worst morning joint stiffness in the past 7 days
	[0 (no joint stiffness)—10 (extreme joint stiffness)]		[0 (no joint stiffness)—10 (extreme joint stiffness)]

*PGIR,* patient global impressions of remission

^a^Although few patients experienced this symptom during the 24-h recall period, this item was retained based on clinical importance for those that experienced it

^b^Item added after first round of interviews

### Patient and clinician concept debriefing

Most patients (86%–100%) demonstrated an understanding of the instructions provided for completing the Crohn’s Disease Diary. In general, patients reported that the items retained following 3 rounds of interviews were easy to understand. When asked if they would be able to complete the Crohn’s Disease Diary daily for one year, 23/27 (85.2%) reported that this would be easy to do and that it would be possible to recall their symptoms over the 24-h and 7-day recall periods. Overall, the electronic format of the diary was deemed acceptable.

Revisions were made iteratively to items in the Crohn’s Disease Diary following each round of interviews, with consideration of patient understanding, conceptual overlap among items, and the translatability of items (following the third round of interviews). An example of a change made to improve understanding was to clearly define “loose or liquid stools.” Due to conceptual overlap of terms, the abdominal pain item was modified to clarify that “abdominal pain” included cramping. Item relevance was also considered after each round of interviews. For example, although few patients reported blood in bowel movements or tenesmus within a given 24-h recall period, these items were retained since most patients experience these symptoms over a longer period of time (i.e., during a clinical trial in which a PRO measure is used). The recall period for the item assessing sleep disturbance due to needing a bowel movement was updated to “in the past 24 h” from “during the night” to be inclusive of alternate work/sleep patterns (as with shift workers), but no other changes were made to recall periods as they were well understood by patients and considered to be appropriate. A nausea item was added following a patient suggestion in the first round of interviews, and an item pertaining to passing gas was removed from the diary due to patient feedback and concern about the feasibility of patients accurately recalling the frequency/severity of this symptom. Clinicians also suggested a lack of clinical importance in assessing gas. Additionally, items relating to difficulty sleeping and general body pain were removed due to redundancy and ambiguity, respectively.

## Discussion

To accurately reflect the patient experience, patients should be involved in the development of measures to be used in clinical trials assessing patient responses to new drug therapies in Crohn’s disease. At the inception of this study, no PRO measures developed according to these standards were available for use that had demonstrated content and psychometric validity. Thus, the aim of this study was to address this unmet need.

The development of the Crohn’s Disease Diary was based on patient and clinician concept elicitation and cognitive debriefing interviews, and informed by a systematic literature review [9]. The concept elicitation questioning identified core symptoms commonly referenced in the literature and extraintestinal symptoms which are experienced by 20% to 40% of patients over the course of their disease [20]. Additional symptoms experienced by a small proportion of the sample were also identified. The final conceptual model indicated that these symptoms have a broad impact on physical functioning, activities of daily living, sleep, and emotional and social functioning. The final 17-item Crohn’s Disease Diary, comprising items phrased with patient-friendly language, provides an assessment of these core symptoms and key impacts over a 24-h recall period and the extraintestinal symptoms over 7 days.

Previously developed instruments (CDAI and IBDQ) have been criticized for lack of standardization of terms (i.e., loose or liquid bowel movements), lack of reproducibility, significant interobserver error, and the use of response scales that do not comply with modern psychometric theory [12, 13]. Although these traditional scales have widely been used in clinical trials, they were developed before regulatory authorities provided guidance on the development of PROs. The 2-stage qualitative research process described herein is consistent with FDA guidance and similar to that used for another recently developed daily PRO measure, the Crohn’s Disease Patient-Reported Outcome Signs and Symptoms (CD-PRO/SS) measure, which became available during the course of this study [21]. The development of both the CD-PRO/SS and the Crohn’s Disease Diary included open-ended, patient-focused concept elicitation questioning to ensure the content validity of the items, followed by patient cognitive interviews. As such, many of the same symptoms, determined to be relevant and important to patients with Crohn’s disease, are captured by both measures. Initial quantitative assessment of the measurement properties of the CD-PRO/SS has been completed [21]. The CD-PRO/SS includes 6 separate modules which can be used separately or in combination. Specific assessment needs may dictate which instrument is more suited. For both instruments, further assessment of the reliability, validity, and importantly, responsiveness in an interventional setting is required to support future use of these measures to assess primary and key secondary endpoints in randomized controlled trials.

In the current study, patient understanding of the concepts “flare” and “remission” were explored since, based on the conceptual model, these are well-recognized concepts and remission is often a primary endpoint in clinical trials. Patient responses were variable in terms of how they defined these terms, and patients’ definitions may not be aligned with common understanding by clinicians. For example, small numbers of patients suggested that the emergence of a specific symptom such as fever signaled a flare, whereas clinicians might consider symptomatology more broadly. Although remission is an important goal for patients, this item was not retained in the final version of the Crohn’s Disease Diary due to the variability of patient definitions of remission.

Patient responses describing impacts to ADLs, work, and school were included in the conceptual model but were also variable. Consequently, it was determined that measuring social and emotional impacts of disease, including the psychological effects such as symptoms of depression and anxiety (which may have many confounding factors) may be best done using instruments with a specific focus on these concepts. In addition, the Crohn’s Disease Diary was developed to align with regulatory guidelines, with a focus on product labeling [1, 2]. It is important to note that a measure that comprehensively assesses all HRQoL impacts could be of value for some research purposes (e.g., a real-world study to capture humanistic burden) or in clinical practice. However, for assessments used in clinical trials that must be completed daily for a number of weeks/months, it is important to assess core symptoms and to balance the comprehensiveness of the instrument with considerations of patient burden. Thus, although additional impacts of disease were explored, it was decided that only items focusing on core symptoms and key impacts thought to be the most important for assessing the effectiveness of treatments and that may be measured as primary or secondary endpoints in a trial should be retained in the final version of the Crohn’s Disease Diary.

Although there is great heterogeneity among patients’ experiences of Crohn’s disease and it is possible that some aspects of the patient experience may not have been captured in the data, all symptoms and impact domains emerged spontaneously within the first two groups of interviews, indicating that conceptual saturation was achieved early on in data collection. However, this study was conducted within the USA and, therefore, cultural differences in patients’ perception of disease may not be represented.

## Conclusion

This study describes the patient experience of Crohn’s disease and the development of a conceptual model and a daily PRO measure for the assessment of Crohn’s disease symptoms and impacts. The Crohn’s Disease Diary provides conceptual coverage with relevant, easy-to-understand items appropriate for use in future drug development programs.

## Supplementary Information

Below is the link to the electronic supplementary material.

Supplementary file1 (DOCX 41 KB)

## Data Availability

Anonymized individual participant data and study documents can be requested for further research from www.clinicalstudydatarequest.com.

## Code availability

N/A.
