# Supplementary material for: A qualitative study to explore the symptoms and impacts of Crohn’s disease and to develop the Crohn’s Disease Diary
Source: Qual Life Res. 2022 Sep 2;32(1):209–23. doi: 10.1007/s11136-022-03233-9 (PMC9829647; doi:10.1007/s11136-022-03233-9)
Supplement: Supplementary file 1 — Supplementary file1 (DOCX 41 KB) [file 11136_2022_3233_MOESM1_ESM.docx]

**Supplemental Materials**

***Disease Severity Categories***

Remission or mild disease was defined as a 7-day average stool frequency score of ≤3 or a 7-day average abdominal pain score of ≤1 in the 3 months prior to screening, and moderate to severe was defined as a 7-day average stool frequency score of ≥4 and/or a 7-day average abdominal pain score of ≥2.

***Detailed Eligibility Criteria***

Inclusion criteria included US residency, cognitive abilities to participate in a 90-minute interview, spoken and written English language fluency, and access to a telephone or computer.

Patients with physical or psychological conditions that, in the opinion of the physician might affect their ability to provide consent, comply with study requirements, or confound study results, were excluded. For example, physical limitations such as limited eyesight or psychological conditions such as attention deficit disorders may have been considered exclusionary by clinicians since reading items in ePRO format was required and it was necessary for patients to maintain concentration for 90 minutes during interviews. If the physician did not think a patient’s condition would have an impact on study results, they were included (e.g., n=3 participants had depression). Additionally, based on clinician judgement, patients with diseases that had overlapping symptoms that may cause difficulty discerning only symptoms and experiences related to Crohn’s disease were excluded.

Eligibility criteria for this study were also based on key exclusion criteria from clinical trials for drugs aimed at treating luminal inflammation. Patients were also excluded if they had an endoscopic procedure within 7 days of screening; a diagnosis of indeterminate colitis, unclassified IBD, infectious colitis, ischemic colitis, or ulcerative colitis; symptomatic strictures/stenoses, short bowel syndrome, or perianal abscess; an ileostomy or colostomy; or bowel surgery within 6 months of screening. Patients currently enrolled in or previously enrolled in a clinical trial within 6 months of screening were not eligible.

[1] Guidance for Industry Patient Reported Outcomes: Use in Medical Product Development to Support Labeling Claims, in: F.a.D. Administration (Ed.) US Department of Health and Human Services, 2009.

[2] Guideline on the development of new medicinal products for the treatment of Crohn’s Disease, in: C.f.M.P.f.H. Use (Ed.) European Medicines Agency, 2016.
